# Patterns of care for non‐metastatic castration‐resistant prostate cancer: A population‐based study

**DOI:** 10.1002/bco2.158

**Published:** 2022-05-18

**Authors:** Shawn Malone, Christopher J. D. Wallis, Richard Lee‐Ying, Naveen S. Basappa, Ilias Cagiannos, Robert J. Hamilton, Ricardo Fernandes, Cristiano Ferrario, Geoffrey T. Gotto, Scott C. Morgan, Christopher Morash, Tamim Niazi, Krista L. Noonan, Ricardo Rendon, Sebastien J. Hotte, Fred Saad, Anousheh Zardan, Brendan Osborne, Katherine F. Y. Chan, Bobby Shayegan

**Affiliations:** ^1^ The Ottawa Hospital University of Ottawa Ottawa Ontario Canada; ^2^ Mount Sinai Hospital, University of Toronto Toronto Ontario Canada; ^3^ Tom Baker Cancer Centre Calgary Alberta Canada; ^4^ Cross Cancer Institute University of Alberta Edmonton Alberta Canada; ^5^ Princess Margaret Cancer Centre University of Toronto Toronto Ontario Canada; ^6^ London Health Sciences Centre Western University London Ontario Canada; ^7^ Jewish General Hospital McGill University Montreal Quebec Canada; ^8^ Southern Alberta Institute of Urology University of Calgary Calgary Alberta Canada; ^9^ BC Cancer Agency University of British Columbia Surrey British Columbia Canada; ^10^ Queen Elizabeth II Health Sciences Centre Dalhousie University Halifax Nova Scotia Canada; ^11^ Juravinski Cancer Centre McMaster University Hamilton Ontario Canada; ^12^ Centre Hospitalier de l'Université de Montréal University of Montreal Montreal Quebec Canada; ^13^ Medical Affairs Janssen Inc Toronto Ontario Canada; ^14^ St. Joseph's Healthcare McMaster University Hamilton Ontario Canada

**Keywords:** androgen deprivation therapy, imaging, non‐metastatic castration‐resistant prostate cancer, PSA testing, real‐world evidence

## Abstract

**Objectives:**

To describe patterns of practice of PSA testing and imaging for Ontario men receiving continuous ADT for the treatment of non‐metastatic castration‐resistant prostate cancer (nmCRPC).

**Patients and Methods:**

This was a retrospective, longitudinal, population‐based study of administrative health data from 2008 to 2019. Men 65 years and older receiving continuous androgen deprivation therapy (ADT) with documented CRPC were included. An administrative proxy definition was applied to capture patients with nmCRPC and excluded those with metastatic disease. Patients were indexed upon progression to CRPC and were followed until death or end of study period to assess frequency of monitoring with PSA tests and conventional imaging. A 2‐year look‐back window was used to assess patterns of care leading up to CRPC as well as baseline covariates.

**Results:**

At a median follow‐up of 40.1 months, 944 patients with nmCRPC were identified. Their median time from initiation of continuous ADT to CRPC was 26.0 months. 60.7% of patients had their PSA measured twice or fewer in the year prior to index, and 70.7% patients did not receive any imaging in the year following progression to CRPC. Throughout the study period, 921/944 (97.6%) patients with CRPC progressed to high‐risk (HR‐CRPC) with PSA doubling time ≤ 10 months, of which more than half received fewer than three PSA tests in the year prior to developing HR‐CRPC, and 30.9% received no imaging in the subsequent year.

**Conclusion:**

PSA testing and imaging studies are underutilized in a real‐world setting for the management of nmCRPC, including those at high risk of developing metastatic disease. Infrequent monitoring impedes proper risk stratification, disease staging and detection of treatment failure and/or metastases, thereby delaying the necessary treatment intensification with life‐prolonging therapies. Adherence to guideline recommendations and the importance of timely staging should be reinforced to optimize patient outcomes.

## 1 INTRODUCTION

Prostate cancer is the second most common cancer in men worldwide.^1^ There was an estimated 1.4 million new cases of prostate cancer worldwide in 2020, making up over 15% of all new cancer cases in men.^2^ The majority of patients with prostate cancer present with early stage at diagnosis^3^ for which local therapy is often curative. However, more than 20% of patients experience biochemical recurrence, indicated by rising prostate‐specific antigen (PSA) levels,^4^, ^5^ for whom androgen deprivation therapy (ADT) is frequently administered.^6^ Castration‐resistant prostate cancer (CRPC) represents an advanced disease state with increased likelihood of metastases.^7^, ^8^ Both non‐metastatic (nmCRPC) and metastatic (mCRPC) states require more intensive treatment and monitoring^9^; thus, timely identification of this state is a priority for optimal management of prostate cancer.

Regular PSA monitoring is essential for the detection of biochemical recurrence following local therapy and CRPC among men on ADT. Frequent imaging enables identification of the transient nmCRPC state prior to visualization of metastases. PSA monitoring and imaging are particularly important for identifying progression as men may be asymptomatic in these disease states. Further, regular monitoring of PSA levels enables precise calculations of PSA doubling time (PSADT), which is useful for identifying patients with high risk for metastases (i.e. PSADT ≤ 10 months). Timely identification of nmCRPC patients, particularly high‐risk nmCRPC, and regular imaging for the detection of metastatic disease enable prompt intensification of systemic therapy to delay metastases and improve survival.^10^, ^11^, ^12^, ^13^

Most clinical trials conducted in nmCRPC patients to date have focused predominantly on high‐risk patients,^10^, ^11^, ^12^, ^13^ and the use of these diagnostic tools to identify CRPC and its progression to more advanced states in real‐world practice has not been thoroughly examined. The objective of this study was to describe patterns of practice for Ontario men receiving continuous ADT for the treatment of CRPC. Specifically, this analysis focused on use of PSA testing and imaging, PSA doubling times following development of CRPC and use of ADT and antiandrogens prior to initiating treatment for metastatic disease.

## 2 METHODS

### 2.1 Study design

This was a retrospective, longitudinal, population‐based study using administrative health data in Ontario, Canada. Ontario makes up approximately 40% of the national population in Canada, with a provincial population size of almost 14.7 million. The cohort included Ontario men aged 66 and older diagnosed with prostate cancer between 1 April 2008 and 31 December 2019, who were receiving continuous ADT treatment, had documented castration resistance and had presumed non‐metastatic disease at index (see specific criteria in Section 2.3). The cohort was limited to patients 66 and older to ensure all patients had comprehensive pharmaceutical coverage via the Ontario Drug Benefit, which becomes available to Ontario residents at 65 years of age. Patients were indexed on the date of castration resistance (i.e., the earliest date of meeting inclusion criteria a, b, and c as stated in Section 2.3) and followed up until the earliest date of death or end of the study period (31 December 2019). A 2‐year look‐back window from index date was used for baseline covariate capture.

### 2.2 Data source

This study used administrative health service records held by the Institute for Clinical and Evaluative Studies (IC/ES), which captures publicly insured healthcare touch points of Ontarians through multiple linked datasets. As all patient data are anonymized, IC/ES has statutory authority to conduct health services research without consent; thus, patient consent was waived. The study received ethics approval from Advarra IRB (Pro00037601).

Data held by IC/ES are collected at the record level, and datasets are linked at the patient level, allowing for longitudinal analysis. This study utilized the following linked datasets: Ontario Cancer Registry (OCR), Registered Persons Database (RPD), Continuing Care Reporting System (CCRS), Ontario Congestive Heart Failure dataset (CHF), Ontario Chronic Obstructive Pulmonary Disease dataset (COPD), Discharge Abstract Database (DAD), Ontario Hypertension dataset (HYPER), National Ambulatory Care Reporting System (NACRS), Ontario Diabetes Dataset (ODD), Ontario Health Insurance Plan Claims database (OHIP), Ontario Myocardial Infarction Dataset (OMID), Ontario Laboratories Information System (OLIS), New Drug Funding Program (NDFP) and Cancer Activity Level Reporting (ALR).

### 2.3 Inclusion and exclusion criteria

The study cohort included Ontario men with presumed nmCRPC that met the following inclusion criteria: diagnosis of prostate cancer between 1 April 2008 and 31 December 2019; aged ≥ 66 years at diagnosis; history of ADT prescription from 30 days prior to prostate cancer diagnosis onwards to end of study period; continuous ADT use, defined as gaps between the anticipated end of ADT effect (prescription date + duration of specific formulation administered) and the next prescription of no more than 30 days; and diagnosis of castration resistance, defined by the following: (a) total PSA level ≥ 2 ng/mL and at least 25% higher than nadir level after at least 1 year of first continuous ADT; (b) second total PSA test at least 3 weeks following the PSA test meeting criterion ‘a’; and (c) total testosterone level < 1.7 nmol/L within 6 months of total PSA level in criteria ‘a’ or ‘b’ after at least 1 year of first continuous ADT prescription.

Although dates of imaging studies are captured at IC/ES, imaging results to determine the presence of metastases are not; thus, we established and validated an administrative proxy definition to distinguish patients with nmCRPC and mCRPC. To be classified as having nmCRPC on the date of biochemical evidence of CRPC, patients must have fulfilled the following criteria: on ADT or history of surgical castration; minimum 1 year of ADT use; and PSA < 20 ng/mL within 90 days prior to the initiation of ADT.

The algorithm used to distinguish nmCRPC and mCRPC patients in the IC/ES data was validated through a collaboration with Alberta Health Services (AHS). Within the AHS dataset, we used hand chart abstraction to determine whether patients had metastasis (mCRPC) or not (nmCRPC) on the date of developing CRPC. Using an overall sample of 866 patients in the AHS dataset (of whom 150 were classified by chart abstraction as nmCRPC), the administrative nmCRPC definition used in the current study was assessed for its sensitivity and specificity. In operationalizing our administrative proxy, we prioritized specificity over sensitivity, as the priority was to ensure mCRPC patients were correctly distinguished and screened out of the cohort, rather than to include as many nmCRPC patients as possible. Using the administrative definition of nmCRPC patients (i.e. by history of local therapy, PSA < 20 ng/mL prior to initiating ADT and time on ADT > 1 year), nmCRPC patients were detected with 53.3% sensitivity and 80.3% specificity, producing a positive predictive value (PPV) of 36.2% and a negative predictive value (NPV) of 89.1%.

Modifications of these definitions were then tested to check for meaningful improvements in predictive ability of the algorithm (detailed in Table S1). Removal of the local therapy or time on ADT criteria produced poorer PPVs and NPVs. Decreasing the PSA threshold to 5, 10 or 15 ng/mL produced a smaller area under the curve (AUC) for the predictive definition, and increasing the PSA threshold to 25, 30 or 50 ng/mL produced negligible improvements in the AUC. The final analysis was thus conducted using the original administrative nmCRPC definition, unchanged.

Patients were excluded if they met any of the following exclusion criteria: female sex; age < 66 years at diagnosis; concomitant radiation therapy with ADT, defined as ADT prescription within 1 month prior to and 3 months post first radiotherapy; PSA‐recommended value > 20 ng/mL within 90 days prior to first continuous ADT prescription; metastatic disease, defined as best stage of IV; missing or invalid identification with ICES key number (IKN); not eligible for OHIP coverage during 2 years prior to diagnosis; or missing data for key exposure for analysis.

### 2.4 Outcomes

This analysis described patterns of progression to the development of CRPC (i.e. the index date) and high‐risk CRPC (i.e. detection of PSADT < 10 months). Specifically, the following outcomes were described for progression to CRPC: time from ADT initiation to index, number of PSA tests prior to index and distribution of imaging studies (including bone scan, computed tomography [CT] or magnetic resonance imaging [MRI]) prior to index. For patients who progressed to high‐risk CRPC (HR‐CRPC), the following outcomes are described: time from CRPC index to HR‐CRPC, number of PSA tests and type of imaging studies prior to detection of HR‐CRPC. In addition, distribution of PSADT and median PSA value after reaching PSADT < 10 months are described for HR‐CRPC patients.

Lastly, for patients who initiated mCRPC treatments, the analysis describes the proportion of patients receiving LHRH agonists or antagonists plus antiandrogens for at least 6 months prior to mCRPC treatment. All outcomes were stratified by geographic location, defined by local health integration network (LHIN). Ontario is made up of 14 LHINs that are responsible for administration of public healthcare services in their respective geographic regions.

### 2.5 Covariates

A 2‐year look‐back window from index date was used for baseline covariate capture and included socio‐demographic information (age, socio‐economic quintile and geographic location), health status (Charlson comorbidity index [CCI], general practitioner [GP] visits and hospitalizations and history of comorbidities) and disease characteristics at index (PSA value, disease stage, Gleason score and prior local treatment).

### 2.6 Data analysis

Descriptive statistics were used to summarize baseline characteristics and outcomes. Categorical variables were summarized as counts and proportions (%), and continuous variables are summarized as medians and interquartile ranges (IQR). Multinomial logistic regression models were used to evaluate the statistical association between geographic location (i.e. LHIN) and outcomes while accounting for covariates as listed above. *P*‐values less than 0.05 were considered statistically significant.

## 3 RESULTS

### 3.1 Patient demographics and baseline characteristics

944 patients (age 66 years and older) with presumed nmCRPC were identified within the ICES databases between April 2008 and December 2019, with a median follow‐up time of 40.1 months (IQR 24.6–55.2 months). The study population had a median age of 75 years (IQR 71–81 years) and was otherwise healthy with a median CCI of 0 (IQR 0–0) (Table 1). For their baseline healthcare utilization in the year prior to index, patients visited their GP a median of seven times (IQR 4–11), and 110 (11.7%) patients had been hospitalized. There was an approximately equal distribution across socio‐economic quintiles, and 803 (85.1%) men lived in a non‐rural setting.

**TABLE 1:** Baseline characteristics (*n* = 944)

Characteristics, *n* (%), mean (SD) or median (IQR)	Total (*N* = 944)
Age	
Median (IQR), years	75 (71–80)
Socio‐economic status and rurality	
Quintile 1	176 (18.6%)
Quintile 2	200 (21.2%)
Quintile 3	176 (18.6%)
Quintile 4	189 (20.0%)
Quintile 5	203 (21.5%)
Rural	141 (14.9%)
Medical care and comorbidity	
Comorbidity (Charlson index)	
Median (IQR)	0 (0–0)
Number of GP visits in year prior to diagnosis	
Median (IQR)	7 (4–11)
Any hospitalization in year prior to diagnosis	110 (11.7%)
Ever LTC resident	1–5^a^
Diabetes	94 (10.0%)
History of MI	24 (2.5%)
History of CVA	8 (0.8%)
History of CHF	52 (5.5%)
History of COPD	52 (5.5%)
History of HTN	153 (16.2%)
History of arrhythmia	8 (0.8%)
History of dementia	41 (4.3%)
History of liver disease	1–5^a^
History of renal disease	67 (7.1%)
Prostate cancer characteristics	
PSA at index	
PSA test	936 (99.2%)
Median (IQR)	4 (3–9)
Stage at diagnosis	
I	17 (1.8%)
II	717 (76.0%)
III	210 (22.2%)
Biopsy Gleason Score	
<7	27 (2.9%)
=7	197 (20.9%)
>7	376 (39.8%)
Unknown	344 (36.4%)
Treatment prior to index date	
Local therapy between PC diagnosis and index date, *n* (%)	353 (37.4%)
Time in months from local treatment to index date, median (IQR)	41.0 (23.3–58.5)
Follow‐up time	
Follow‐up time in months, median (IQR)	40.1 (24.6–55.2)

^a^ Denotes cases where a range of patients involved has been provided to avoid the potential for patient identification due to confidentiality.

In terms of disease characteristics, the median PSA at the time of CRPC was 4 ng/mL (IQR 3–9 ng/mL). The majority of patients (717, 76.0%) were Stage II at diagnosis; among 600 (64.3%) patients with complete biopsy histology, 573 (60.7%) patients had a biopsy Gleason score ≥ 7. 353 (37.4%) patients had received local therapy (i.e. radiotherapy or radical prostatectomy) between prostate cancer diagnosis and index date; of these patients, median time from local therapy to CRPC index was 41.0 (IQR 23.3–58.5) months.

Median time from continuous ADT initiation to CRPC index was 26.0 (IQR 17.0–43.5) months across the total cohort; 432 (45.8%) patients developed CRPC within 24 months of starting ADT. These results were consistent across the LHINs (*p* = 0.39 and *p* = 0.58, respectively) (Table S2).

### 3.2 Progression to CRPC

#### 3.2.1 PSA testing

In the 12 months prior to developing castration resistance, 229 (28.7%) had their PSA measured only once, 255 (32.0%) patients measured twice, 175 (21.9%) measured thrice and 139 (17.4%) measured at least four times. In the 12 months prior to developing HR‐CRPC, 157 (17.3%) had their PSA measured only once, 326 (35.9%) patients measured twice, 234 (25.8%) measured thrice, and 190 (20.9%) measured at least four times. These results did not vary significantly by region nor with other baseline covariates (Table S4). Upon development of HR‐CRPC, over two‐thirds of patients were detected with an initial PSA doubling time of less than 5 months (Table S5), and their median PSA values ranged from 9 (4–22) ng/mL at PSADT <3 months to 6 (4–16) ng/mL at PSADT 3–5 months (Table S6).

#### 3.2.2 Imaging

Most patients (667, 70.7%) did not receive any imaging in the first 3 months following CRPC index. Within the 12 months following development of castration resistance, 331 (35.1%) patients still had no documented imaging (Table 2). Again, no significant interaction was detected across regions nor with other baseline covariates.

**TABLE 2:** Number of patients receiving CT/MRI and/or bone scan, or no imaging, following development of CRPC (*n* = 944) and HR‐CRPC (*n* = 921)

Imaging studies in the 12 months post‐CRPC index (*n* = 944)			
Ontario LHINs (de‐identified)	No imaging	CT/MRI or bone scan	CT/MRI and bone scan
A	9 (25.7%)	7 (20.0%)	19 (54.3%)
B	18 (25.7%)	12 (17.1%)	40 (57.1%)
C	25 (45.5%)	12 (21.8%)	18 (32.7%)
D	52 (44.8%)	19 (16.4%)	45 (38.8%)
E	11–15^a^	1–5^a^	15 (48.4%)
F	26 (39.4%)	11 (16.7%)	29 (43.9%)
G	26 (33.3%)	18 (23.1%)	34 (43.6%)
H	42 (33.6%)	25 (20.0%)	58 (46.4%)
I	41 (40.6%)	21 (20.8%)	39 (38.6%)
J	19 (36.5%)	13 (25.0%)	20 (38.5%)
K	17 (19.5%)	14 (16.1%)	56 (64.4%)
L	11–15^a^	3–7^a^	24 (57.1%)
M	22 (33.8%)	9 (13.8%)	34 (52.3%)
N	6–10^a^	1–5^a^	10 (47.6%)
All LHINs	331 (35.1%)	172 (18.2%)	441 (46.7%)
*p*‐value	0.059		

Imaging studies in the 12 months following PSADT ≤10 months among HR‐CRPC patients (*n* = 921)			
Ontario LHINs (de‐identified)	No imaging	CT/MRI or bone scan	CT/MRI and bone scan
A	10 (29.4%)	8 (23.5%)	16 (47.1%)
B	15 (22.4%)	11 (16.4%)	41 (61.2%)
C	22 (40.0%)	14 (25.5%)	19 (34.5%)
D	42 (37.5%)	26 (23.2%)	44 (39.3%)
E	9–13^a^	1–5^a^	17 (54.8%)
F	25 (39.7%)	11 (17.5%)	27 (42.9%)
G	21 (28.0%)	17 (22.7%)	37 (49.3%)
H	31 (25.4%)	23 (18.9%)	68 (55.7%)
I	34 (34.7%)	21 (21.4%)	43 (43.9%)
J	13 (26.0%)	15 (30.0%)	22 (44.0%)
K	17 (19.5%)	14 (16.1%)	56 (64.4%)
L	12 (28.6%)	8 (19.0%)	22 (52.4%)
M	23 (35.9%)	11 (17.2%)	30 (46.9%)
N	7–11^a^	3–7^a^	7 (33.3%)
All LHINs	285 (30.9%)	187 (20.3%)	449 (48.8%)
*p*‐value	0.063		

^a^ Denotes cases where a range of patients involved has been provided to avoid the potential for patient identification due to confidentiality.

Of those who were imaged within 1 year of CRPC, the majority received both a CT/MRI and bone scan (441 patients, 46.7% of total cohort), whereas the remainder were imaged with either CT/MRI or bone scan, but not both (172 patients, 18.2% of total cohort) (Table 2).

#### 3.2.3 High‐ versus low‐risk CRPC patients within 6 months of CRPC index

We then stratified patients based on their PSADT as calculated within 6 months of their index date. Among 647 (68.5%) high‐risk patients (i.e. those with PSADT ≤ 10 months), the median number of PSA tests in the year following CRPC index was 4 (IQR 3–6). In contrast, among the 297 (31.5%) low‐risk patients (i.e. those with PSADT > 10 months), a median of 2 (IQR 2–3) PSA tests was performed in the year following CRPC index. The difference between these groups was statistically significant (*p* < 0.001). Similarly, 166 (55.9%) low‐risk patients did not receive any imaging studies done in the year following CRPC index, versus only 165 (25.5%) high‐risk patients (*p* < 0.001) (Table 3).

**TABLE 3:** Number of PSA tests and imaging studies in the year following CRPC, stratified by risk (PSADT >10 or ≤10) within 6 months following CRPC (*n* = 944)

Outcome	Label	Low risk (PSADT > 10)	High risk (PSADT ≤ 10)	Total	*p*‐value
		*N* = 297	*N* = 647	*N* = 944	
PSA tests in 1 year following CRPC index date	Median (IQR)	2 (2–3)	4 (3–6)	3 (2–5)	<0.001
Imaging studies in 1 year following CRPC index date (column percentage)	No imaging	166 (55.9%)	165 (25.5%)	331 (35.1%)	<0.001
	CT/MRI or bone scan	56 (18.9%)	116 (17.9%)	172 (18.2%)	
	Ct/MRI + bone scan	75 (25.3%)	366 (56.6%)	441 (46.7%)	

#### 3.2.4 Progression to mCRPC

At a median follow‐up 40.1 months following development of presumed nmCRPC, 557 (59.0%) patients progressed and started on mCRPC medications. Of these patients, 157 (28.2%) received an LHRH agonist or antagonist and an antiandrogen for at least 6 months prior to initiating mCRPC medications. There was no significant variation with secondary hormonal manipulation across LHINs (*p* = 0.65).

## 4 DISCUSSION

This Canadian real‐world study of patterns of care among patients with presumed non‐metastatic castration‐resistant prostate cancer is the first of its kind in this population. In this large, population‐based cohort, PSA testing and imaging studies were underutilized for the care of Canadian men with nmCRPC following initiation of continuous ADT. In the year prior to progression to CRPC, most patients received two or less PSA tests, and, in the year post‐CRPC development, more than 35% of patients received neither a bone, CT nor MRI scan. Progression to high‐risk disease (i.e. PSADT ≤ 10 months) did not trigger more intensive monitoring; over 50% received two or less PSA tests in the year prior to HR‐CRPC, and 31% received no imaging studies in the subsequent 12 months. No significant interactions were detected across regions nor with baseline covariates. These patterns of monitoring deviate from clinical practice guidelines that recommend PSA testing every 3–4 months, as well as imaging every 3–6 months in patients with PSADT ≤ 10 months and every 6–12 months for those with PSADT > 10 months.^9^ Where imaging frequency was adhered to as per recommendation, mixed imaging modality was utilized (i.e. bone scan and CT/MRI scan) to screen for both bone metastases and lymph node and visceral metastases.

Further, our results show that after initiating continuous ADT, more than 80% of patients subsequently progress to HR‐CRPC in the following year, with an initial PSA doubling time measured at less than 5 months for over two‐thirds of the patients and subsequently progressed to metastatic CRPC within 3 years. The time to progression seen in this study is considerably faster than other estimates of progression to mCRPC in the literature^14^, ^15^; however, it warrants to note that CRPC was an inclusion criterion for this study. Thus, those patients who initiated ADT but had not yet progressed to CRPC were excluded. This will serve to underestimate the true median time to CRPC and at least partly explain the discrepancy between these results and other estimates in the literature.

Overall, the patterns of care reported in this study indicate that patients are under‐monitored and under‐managed leading up to and beyond the development of CRPC, which can lead to gaps in quality care and negatively impact patient outcomes. First, infrequent PSA monitoring following initiation of continuous ADT may cause a delay in diagnosing castration resistance and thus delay time to treatment intensification. Further, infrequent PSA testing does not allow for accurate determination of the PSADT, impeding risk categorization and identification of biochemical failures in a timely manner, and thereby does not trigger appropriate additional imaging studies for proper staging, nor detection of disease progression. As such, patients may progress from castration‐sensitive disease seemingly directly to mCRPC, without the transitory nmCRPC state in between. Clinically important treatment options are now available for nmCRPC patients experiencing biochemical failure before the emergence of radiographic progression. Recently, the androgen receptor signalling inhibitors (apalutamide, enzalutamide and darolutamide) have been proven to significantly delay metastasis and prolong survival among patients with high‐risk nmCRPC disease.^10^, ^11^, ^12^, ^13^ Though this previously unmet need has been filled, these high‐risk nmCRPC patients cannot be optimally treated if they are not identified in a timely manner. Despite the fact that PSMA PET imaging has gradually been incorporated in many countries in the staging and management of prostate cancer, its access is currently restricted to clinical trials in the single‐payer healthcare system in Ontario Canada. The relevance to our findings should not change with different imaging modalities as we aim to describe the pattern of practice for this population where Level 1 evidence exists to improve outcome with traditional imaging (i.e. bone and/or CT scans).

The real‐world evidence presented in the current study addresses an important gap in the literature. Clinical trials in nmCRPC populations mandate rigorous monitoring, allowing for precise treatment and management based on staging and progression. Our results have highlighted a significant gap between clinical trial practices and real‐world patterns of care with respect to monitoring. Understanding real‐world practice is crucial for contextualizing clinical trial outcomes, identifying factors that broaden the gap between efficacy and effectiveness and aid in pinpointing areas for improvement in practices. That said, use of real‐world data has its own limitations. First, this population‐based database lacked data on imaging results, thus limiting the ability to accurately identify the timing of metastasis development. This necessitated the use of a proxy definition that has been validated through an independent database to select for greater specificity over sensitivity. Second, in order to minimize variability in insurance coverage and access to treatment, this cohort was limited to patients 65 and older in a single‐payer healthcare system. These results may not be generalizable to the younger cohort or patients outside of Ontario, Canada. Third, this study selected for prostate cancer patients with advanced disease. Our cohort only included patients on continuous ADT with documented castration resistance, and results may not generalize to patients who have not initiated continuous ADT or who do not develop castrate resistance. Finally, this study was conducted over a period with limited treatment options for HR‐CRPC, so it is plausible that poor adherence to monitoring is due to the lack of change in treatment course at the time regardless of monitoring and findings.

The treatment landscape for nmCRPC has evolved considerably. This study has demonstrated a potential barrier to successfully incorporating available treatments for nmCRPC into real‐world practice where patients' progression to metastatic disease can be delayed and survival can be prolonged. Thus, close monitoring with appropriate utilization of PSA testing and imaging is warranted to improve risk stratification and detection of progression and ultimately improve patient outcomes in order to provide quality care.

## CONFLICT OF INTEREST

**SM:** Honoraria (Astellas Pharma, Bayer, Janssen, Sanofi); Travel, Accommodations, Expenses (Sanofi, TerSera). **CJDW:** Consulting or Advisory role: (Janssen Oncology). **RLY:** Honoraria (Janssen); Consulting or Advisory role (Amgen, Bayer, Celgene, Ipsen, Janssen, Pfizer, Sanofi); Speakers' Bureau (Amgen, Celgene, Janssen); Research Funding (Sanofi). **NSB:** Honoraria (Astellas Pharma, Eisai, Ipsen, Janssen, Merck, Pfizer); Consulting or Advisory role (Astellas Pharma, AstraZeneca, Bayer, Bristol‐Myers Squibb, Eisai, Ipsen, Janssen, Merck, Pfizer, Roche Canada); Travel, Accommodations, Expenses (Eisai). **IC:** Honoraria (Abbvie, Ferring, Janssen); Travel, Accommodations, Expenses (TerSera). **RJH:** Honoraria (Abbvie, Amgen, Astellas Pharma, Bayer, Janssen); Research Funding (Bayer, Janssen); Travel, Accommodations, Expenses (Janssen). **RF:** Honoraria (Bayer, Merck, Pfizer); Consulting or Advisory role (Bayer, Janssen, Novartis Canada Pharmaceuticals Inc, Pfizer); Travel, Accommodations, Expenses (Janssen). **CF:** Honoraria (Astellas Pharma, AstraZeneca, Bayer, Lilly, Merck, Novartis, Pfizer); Consulting or Advisory role (AstraZeneca, Odonate Therapeutics, Pfizer, Roche); Speakers' Bureau (Janssen); Research Funding (Astellas Pharma, AstraZeneca, Immunomedics, Janssen, Merck, Novartis, Pfizer, Roche, Sanofi, Seattle Genetics). **GTG:** Honoraria: (Amgen, Astellas Pharma, Bayer, Janssen, Merck); Consulting or Advisory role (Amgen, Astellas Pharma, Bayer, Janssen, Merck); Expert Testimony (Janssen); Travel, Accommodations, Expenses (Janssen). **SCM:** Consulting or Advisory role (Astellas Pharma, Bayer, Janssen, TerSera). **CM:** Consulting or Advisory role (Amgen, AbbVie, Bayer, Astellas, Janssen, TerSera, Knight, Verity, Sanofi). **TN:** Honoraria (Abbvie, Astellas Pharma, Bayer, Janssen Oncology, Sanofi, TerSera); Consulting or Advisory role (Abbvie, Bayer, Janssen Oncology, Sanofi, TerSera); Research Funding (Astellas Pharma, Bayer, Sanofi, TerSera); Travel, Accommodations, Expenses (Janssen Oncology, Sanofi, TerSera). **KN:** Consulting or Advisory role (AstraZeneca, Bristol‐Myers Squibb, Ipsen, anssen Oncology, Merck, Pfizer, Roche Canada, Sanofi); Speakers' Bureau (Merck). **RR**: Advisory role and Honoraria (AbbVie, Amgen, Astellas, AstraZeneca, Bayer, Ferring, Janssen and Sanofi). **SJH:** Honoraria (Astellas Scientific and Medical Affairs Inc, Bayer, Janssen Oncology, Merck); Consulting or Advisory role (Astellas Pharma, AstraZeneca, Bayer, Bristol‐Myers Squibb, Eisai, Ipsen, Janssen, Merck, Pfizer, Roche Canada); Research Funding (Astellas Pharma, AstraZeneca, Ayala Pharmaceuticals, Bristol‐Myers Squibb, Clovis Oncology, Janssen Oncology, Merck, Pfizer, Roche/Genentech, Seattle Genetics, SignalChem); Travel, Accommodations, Expenses (Eisai). **FS:** Honoraria (Abbvie, Astellas Pharma, AstraZeneca, Bayer, Janssen Oncology, Myovant Sciences, Pfizer, Sanofi); Consulting or Advisory role (Abbvie, Astellas Pharma, AstraZeneca/MedImmune, Bayer, Janssen Oncology, Myovant Sciences, Pfizer, Sanofi); Research Funding (Astellas Pharma, AstraZeneca, Bayer, Bristol‐Myers Squibb, Janssen Oncology, Pfizer, Sanofi). **AZ**: Employee of Janssen. **BO:** Employee of Janssen. **KFYC:** Employee of Janssen. **BS:** Honoraria (Abbvie, Astellas Pharma, Bayer, Janssen, Knight Pharmaceuticals, TerSera); Consulting or Advisory role (Abbvie, Astellas Pharma, Bayer, Janssen, Knight Pharmaceuticals, TerSera); Speakers' Bureau (Abbvie, Astellas Pharma, Bayer, Janssen, Knight Pharmaceuticals, TerSera); Research Funding (Astellas Pharma, Janssen, Merck).

## DISCLAIMERS

Dr Wallis had full access to all the data in the study and takes responsibility for the integrity of the data and the accuracy of the data analysis. This study contracted ICES Data & Analytic Services (DAS) and used de‐identified data from the ICES Data Repository, which is managed by ICES with support from its funders and partners: Canada's Strategy for Patient‐Oriented Research (SPOR), the Ontario SPOR Support Unit, the Canadian Institutes of Health Research and the Government of Ontario. Parts of this material are based on data and information provided by Cancer Care Ontario (CCO). Parts of this material are based on data and information compiled and provided by CIHI. The opinions, results and conclusions reported are those of the authors. No endorsement by ICES or any of its funders or partners is intended or should be inferred. The opinions, results, view, and conclusions reported in this paper are those of the authors and do not necessarily reflect those of CCO. No endorsement by CCO is intended or should be inferred. The analyses, conclusions, opinions and statements expressed herein are those of the author, and not necessarily those of CIHI.

## AUTHOR CONTRIBUTIONS

Concept and design: All authors. Acquisition, analysis or interpretation of data: **CJDW**, **KFYC**, **BO**, **SM**, **BS**, **FS**. Drafting of the manuscript: **CJDW**, **KFYC**, **BO**, **SM**, **BS**. Critical revision of the manuscript for important intellectual content: All authors. Statistical analysis: **CJDW**, **KFYC**, **BO**, **SM**, **BS**. Administrative, technical, or material support: **CJDW**, **KFYC**, **AZ**, **BO**. Supervision: **CJDW**, **SM**, **BS**, **FS**.

## Supporting information


**Table S1:** Validation of proxy definition of nmCRPC using AHS dataset
**Table S2:** Time from ADT initiation to development of CRPC (n = 944)
**Table S3:** Time from development of CRPC to HR‐CRPC (n = 921)
**Table S4:** Number of PSA tests in the 12 months prior to HR‐CRPC (n = 907)
**Table S5:** Distribution of initial PSA doubling time after reaching HR‐nmCRPC (i.e., PSADT ≤ 10 months) (n = 921)
**Table S6:** Median PSA values when PSADT ≤ 10 months, by PSADT group (n = 921)

## Data Availability

Health administrative databases for Ontario, Canada, are housed at IC/ES (www.ices.on.ca).
