# Supplementary material for: Patterns of care for non‐metastatic castration‐resistant prostate cancer: A population‐based study
Source: BJUI Compass. 2022 May 18;3(5):383–91. doi: 10.1002/bco2.158 (PMC9349587; doi:10.1002/bco2.158)
Supplement: Supplementary file 1 — Table S1: Validation of proxy definition of nmCRPC using AHS dataset Table S2: Time from ADT initiation to development of CRPC (n = 944) Table S3: Time from development of CRPC to HR‐CRPC (n = 921) Table S4: Number of PSA tests in the 12 months prior to HR‐CRPC (n = 907) Table S5: Distribution of initial PSA doubling time after reaching HR‐nmCRPC (i.e., PSADT ≤ 10 months) (n = 921) Table S6: Median PSA values when PSADT ≤ 10 months, by PSADT group (n = 921) [file BCO2-3-383-s001.docx]

Supplementary Materials

Supplementary Table 1: Validation of proxy definition of nmCRPC using AHS dataset

| Local Therapy | PSA<20 | Time>365 days | Accuracy and Predictive Values |
| --- | --- | --- | --- |
| Yes | Yes | Yes | Sensitivity: 80/150= 53.3%  Specificity: 575/716= 80.3%  PPV: 80/221= 36.2%  NPV: 575/645= 89.1% |
| No | Yes | Yes | Sensitivity: 91/150= 60.7%  Specificity: 483/716= 67.5%  PPV: 91/324= 28.1%  NPV: 483/542= 89.1% |
| Yes | Yes | No | Sensitivity: 85/150= 56.7%  Specificity: 499/716= 69.7%  PPV: 85/302= 28.1%  NPV: 499/564= 88.5% |

AHS = Alberta Health Services

PPV = Positive predictive value

NPV = Negative predictive value

Supplementary Table 2: Time from ADT initiation to development of CRPC (n= 944)

| **Ontario LHINs** | **Eligible patients** | **Months from ADT initiation to CRPC,**  **Median (IQR)** | **CRPC developed within 24 months of ADT initiation, n (%)** |
| --- | --- | --- | --- |
| A | N=35 | 23.5 (17.0-37.0) | 19 (54.3%) |
| B | N=70 | 23.0 (16.0-34.0) | 36 (51.4%) |
| C | N=55 | 26.5 (16.5-46.5) | 24 (43.6%) |
| D | N=116 | 25.5 (18.0-39.5) | 55 (47.4%) |
| E | N=31 | 31.5 (16.0-48.5) | 11 (35.5%) |
| F | N=66 | 24.5 (15.0-44.0) | 32 (48.5%) |
| G | N=78 | 23.0 (15.5-36.5) | 41 (52.6%) |
| H | N=125 | 27.0 (17.0-41.0) | 55 (44.0%) |
| I | N=101 | 24.5 (15.5-45.5) | 48 (47.5%) |
| J | N=52 | 24.5 (15.5-44.5) | 23 (44.2%) |
| K | N=87 | 26.0 (18.0-44.5) | 42 (48.3%) |
| L | N=42 | 28.0 (19.0-45.5) | 17 (40.5%) |
| M | N=65 | 31.0 (19.5-54.0) | 21 (32.3%) |
| N | N=21 | 33.5 (22.0-57.0) | 8 (38.1%) |
| All LHINs | N=944 | 26.0 (17.0-43.5) | 432 (45.8%) |
| p-value |  | 0.390 | 0.578 |

Supplementary Table 3: Time from development of CRPC to HR-CRPC (n= 921)

| **Ontario LHINs** | **Days from CRPC to HR-nmCRPC (n= 921), Median (IQR)** | **HR-nmCRPC developed within 12 months of CRPC index date, n (%)** | **HR-nmCRPC developed within 24 months of CRPC index date, n (%)** |
| --- | --- | --- | --- |
| A | 116.5 (63.0-370.0) | 25 (71.4%) | 31 (88.6%) |
| B | 94.0 (43.0-267.0) | 57 (81.4%) | 66 (94.3%) |
| C | 166.0 (91.0-321.0) | 44 (80.0%) | 51 (92.7%) |
| D | 105.0 (76.5-184.5) | 101 (87.1%) | 109 (94.0%) |
| E | 107.0 (87.0-234.0) | 24 (77.4%) | 28 (90.3%) |
| F | 107.0 (42.0-277.0) | 51 (77.3%) | 58 (87.9%) |
| G | 122.0 (65.0-286.0) | 62 (79.5%) | 71 (91.0%) |
| H | 109.0 (64.0-193.0) | 103 (82.4%) | 118 (94.4%) |
| I | 96.5 (59.0-175.0) | 83 (82.2%) | 89 (88.1%) |
| J | 105.0 (74.0-309.0) | 41 (78.8%) | 48 (92.3%) |
| K | 108.0 (82.0-178.0) | 76 (87.4%) | 84 (96.6%) |
| L | 96.5 (58.0-155.0) | 39 (92.9%) | 41 (97.6%) |
| M | 93.5 (42.5-196.0) | 52 (80.0%) | 61 (93.8%) |
| N | 216.5 (133.5-287.5) | 18 (85.7%) | 19 (90.5%) |
| All LHINs | 107.0 (70.5-211.0) | 776 (82.2%) | 874 (92.6%) |
| p-value | 0.074 | 0.489 | 0.573 |

Supplementary Table 4: Number of PSA tests in the 12 months prior to HR-CRPC (n= 907)

| **Ontario LHINs** | **1** | **2** | **3** | **4+** |
| --- | --- | --- | --- | --- |
| A | 6 (18.2%) | 12 (36.4%) | 6 (18.2%) | 9 (27.3%) |
| B | 9 (13.4%) | 17 (25.4%) | 17 (25.4%) | 24 (35.8%) |
| C | 7 (12.7%) | 23 (41.8%) | 18 (32.7%) | 7 (12.7%) |
| D | 26 (23.4%) | 36 (32.4%) | 25 (22.5%) | 24 (21.6%) |
| E | *4 - 8 | 15 (50.0%) | *1 - 5 | *4 - 8 |
| F | 10 (16.1%) | 21 (33.9%) | 20 (32.3%) | 11 (17.7%) |
| G | 8 (11.1%) | 32 (44.4%) | 16 (22.2%) | 16 (22.2%) |
| H | 23 (19.5%) | 38 (32.2%) | 40 (33.9%) | 17 (14.4%) |
| I | 17 (17.3%) | 34 (34.7%) | 25 (25.5%) | 22 (22.4%) |
| J | 6 (12.0%) | 19 (38.0%) | 12 (24.0%) | 13 (26.0%) |
| K | 19 (21.8%) | 28 (32.2%) | 23 (26.4%) | 17 (19.5%) |
| L | 6 (14.6%) | 13 (31.7%) | 12 (29.3%) | 10 (24.4%) |
| M | 11 (17.5%) | 28 (44.4%) | 12 (19.0%) | 12 (19.0%) |
| N | *1 - 5 | 10 (50.0%) | *1 - 5 | *1 - 5 |
| All LHINs | 157 (17.3%) | 326 (35.9%) | 234 (25.8%) | 190 (20.9%) |
| P-value | 0.361 | | | |

* Denotes cases where a range of patients involved has been provided to avoid the potential for patient identification due to confidentiality.

Supplementary Table 5: Distribution of initial PSA doubling time after reaching HR-nmCRPC (i.e., PSADT ≤ 10 months) (n= 921)

|  | **Initial PSA doubling time,**  **n (%)** | | | | |
| --- | --- | --- | --- | --- | --- |
| **Ontario LHINs** | **< 3 months** | **3 – 5 months** | **5.1 – 7 months** | **7.1 – 9 months** | **9.1 – 10 months** |
| A | 12 (35.3%) | 13 (38.2%) | *4 - 8 | *1 - 5 | 0 (0.0%) |
| B | 34 (50.7%) | 17 (25.4%) | 9 (13.4%) | *1 - 5 | *1 - 5 |
| C | 22 (40.0%) | 11 (20.0%) | 14 (25.5%) | *4 - 8 | *1 - 5 |
| D | *39 - 43 | 32 (28.6%) | 24 (21.4%) | 9 (8.0%) | *4 - 8 |
| E | 11 (35.5%) | 9 (29.0%) | *1 - 5 | *1 - 5 | *4 - 8 |
| F | 21 (33.3%) | 22 (34.9%) | 13 (20.6%) | *4 - 8 | *1 - 5 |
| G | 29 (38.7%) | 15 (20.0%) | *11 - 15 | 15 (20.0%) | *1 - 5 |
| H | 46 (37.7%) | 42 (34.4%) | 19 (15.6%) | *7 - 11 | *4 - 8 |
| I | 41 (41.8%) | 24 (24.5%) | 17 (17.3%) | *8 - 12 | *4 - 8 |
| J | 19 (38.0%) | 10 (20.0%) | 7 (14.0%) | 7 (14.0%) | 7 (14.0%) |
| K | 41 (47.1%) | 28 (32.2%) | 10 (11.5%) | *3 - 7 | *1 - 5 |
| L | 17 (40.5%) | 12 (28.6%) | 8 (19.0%) | *1 - 5 | *1 - 5 |
| M | 28 (43.8%) | 11 (17.2%) | 16 (25.0%) | *1 - 5 | *4 - 8 |
| N | *1 - 5 | 8 (38.1%) | 8 (38.1%) | *1 - 5 | *1 - 5 |
| All LHINs | 364 (39.5%) | 254 (27.6%) | 167 (18.1%) | 87 (9.4%) | 49 (5.3%) |
| p-value | 0.022 | | | | |

* Denotes cases where a range of patients involved has been provided to avoid the potential for patient identification due to confidentiality.

Supplementary Table 6: Median PSA values when PSADT ≤ 10 months, by PSADT group (n= 921)

|  | **PSA values (ng/mL),**  **Median (IQR)** | | | | | |  |
| --- | --- | --- | --- | --- | --- | --- | --- |
| **Ontario LHINs** | **< 3 months** | **3 – 5 months** | **5.1 – 7 months** | **7.1 – 9 months** | **9.1 – 10 months** | **Total** | **P-value** |
| A | 16 (7-36) | 7 (3-11) | 6 (1-39) | 10 (4-16) | - | 10 (4-30) | 0.343 |
| B | 7 (3-20) | 7 (4-24) | 6 (3-24) | 12 (6-25) | 11 (5-47) | 7 (4-24) | 0.898 |
| C | 8 (3-17) | 16 (6-26) | 5 (3-12) | 2 (2-8) | 6 (3-9) | 8 (3-16) | 0.103 |
| D | 9 (5-17) | 6 (3-10) | 6 (3-9) | 4 (2-7) | 5 (3-6) | 7 (4-11) | 0.040 |
| E | 10 (4-35) | 6 (4-7) | 6 (4-8) | 5 (2-7) | 6 (2-7) | 6 (3-10) | 0.369 |
| F | 13 (6-37) | 5 (3-13) | 4 (2-11) | 3 (1-4) | 5 (4-7) | 7 (3-17) | 0.284 |
| G | 5 (3-12) | 5 (4-9) | 8 (6-19) | 5 (3-16) | 4 (3-7) | 6 (3-14) | 0.781 |
| H | 7 (4-24) | 8 (4-15) | 5 (3-11) | 8 (2-11) | 8 (5-15) | 7 (4-15) | 0.706 |
| I | 9 (4-13) | 5 (3-13) | 7 (3-17) | 4 (3-5) | 8 (2-8) | 6 (3-13) | 0.267 |
| J | 10 (6-22) | 9 (3-20) | 4 (3-9) | 6 (4-8) | 27 (8-75) | 8 (5-22) | 0.052 |
| K | 6 (4-14) | 8 (5-19) | 4 (4-19) | 3 (2-7) | 11 (4-17) | 6 (4-17) | 0.166 |
| L | 11 (6-27) | 7 (4-25) | 5 (4-7) | 5 (3-7) | 3 (2-4) | 7 (4-22) | 0.070 |
| M | 15 (6-43) | 4 (3-10) | 5 (2-14) | 2 (0-5) | 5 (4-6) | 6 (3-20) | 0.005 |
| N | 1 (1-1) | 18 (5-37) | 10 (5-31) | 7 (4-11) | 8 (8-8) | 9 (5-25) | 0.512 |
| All LHINs | 9 (4-22) | 6 (4-16) | 6 (3-12) | 4 (3-8) | 6 (4-9) | 7 (4-16) | <.001 |
